# Supplementary material for: A snapshot on a journey from frustration to readiness–A qualitative pre-implementation exploration of readiness for technology adoption in Public Health Protection in Ireland
Source: PLOS Digit Health. 2024 Mar 5;3(3):e0000453. doi: 10.1371/journal.pdig.0000453 (PMC10914281; doi:10.1371/journal.pdig.0000453)
Supplement: S2 Text — (DOCX) [file pdig.0000453.s002.docx]

# **S2 Text. Interview guide**

# ***Interview guide for semi-structured key informant interviews***

Topics & Questions for interview

| **Topic** | **Questions** |
| --- | --- |
| **UNDERSTANDING PARTICIPANT PERSPECTIVES (STATUS QUO)** | |
| **Time in the department** | - How long have you worked in the Department of Public Health (DoPH)? - Is this your first role in the HSE (Health Service Executive)? - *Explore any other healthcare related roles where participant might have experienced changes to work practices, or where digital systems may be relevant* |
| **Role in case & incident management**: | - Thinking about the current methods of case and incident management (CIM), can you briefly describe for me your role in this process? |
| **Digital literacy:** | - What parts of your role in CIM involve working with digital systems or computers? - How do you find this part of your work? *Probe - easy or challenging,* Do you like it? - How do you think your colleagues find working with the digital elements of your work? |
| **ESTABLISHING STATUS QUO ON PROJECT IMPLEMENTATION** | |
| **Formal or informal channels of disseminating change information**  **Social needs** - **existence of a peer network** | - How aware are you of the plan for the new CIMS? - How did you become aware of the planned switch to a CIMS - *Explore: communication channels, regular meetings* – how often do you get updates? From whom? Informal or formal channels? From organised groups or private messages? – *is there a social aspect of working with colleagues, or groups that talks about changes to work practices – is there a peer network?* |
| **Personal response to specific change, personal modifiers of feelings about change** | - Can you describe to me how you felt when you became aware of the plan to bring in a new CIMS? What do you think made you feel that way? |
| **Individual concerns/needs** | - Did you have any initial concerns about the change? |
| **Collective modifiers of feelings about change.** | - How did you think your colleagues felt about the change? What do you think made them feel that way? |
| **Personal assessment of the problem the technology or change is addressing** | - What would say are the strengths of the current process of CIM? - What would say are the weaknesses of the current process of CIM? - How do you think other DoPH feel about their current CIM processes? |

| **EXPLORATION OF THE FACTORS FOR CONSIDERATION AND FUTURE USERS NEEDS IN IMPLEMENTATION & UTILISATION OF THE CIMS** | |
| --- | --- |
| **Personal experience of change, lessons to be learnt from previous experiences, facilitation conditions.** | |
|  | - Aside from the design and capabilities of the system, what springs to mind as an important factor for the implementation of this new CIMS? |
| **Communication** | - Who is delivering messages or updates? - *Explore: Channels of communication; Frequency; Is there peer networks?* - Who needs to be kept informed? - What makes communication good or bad? - *Explore: Feedback channels – reciprocal communication* |
| **Training** | - What is your preferred method of learning to use the new system? *Explore: Formats (online, videos, in person):* - *Explore: Permanent resources (handbooks*) - *What works, what doesn’t work?:* Can you describe for me any other methods of learning new technologies or techniques that you found useful. Can you describe for me any other methods of learning new technologies or methods that found were not effective? |
| **Support** | - *Explore methods of providing continued support after implementation (e.g. superuser)* - Day to day training, how to provide it? |
| **Other topics that might arise** | - Time needed - ICT - Leadership |
| **PERCEPTION OF, AND BARRIERS & ENABLERS TO ORGANISATIONAL READINESS & WILLINGNESS** | |
|  | - Thinking of your own department, how ready do you think everyone is switch to a new way of working for CIM? - Could you expand on this, what is making people feel this way (ready/not ready)? - What factors do you feel are currently hampering/supporting readiness for rolling out the new CIMS? - What else can be done to enhance this sense of readiness? - Thinking of the wider network…how ready do you think other DoPH are to make this switch - Could you expand on this, what do think may be making people feel this way (ready/not ready)? - Is it important for you to know that other departments are ready to implement the new system? - Thinking of the wider network of DoPHs, how willing do you think the departments are to switch to working with a standardised method of CIM? - What do you think may be making them feel this way? - What stands out for you as being important to enhance/maintain this willingness? - Reflecting on the DoPHs nationally, how ready do you think the organisation is to bring in this new CIMS? Can you give me more detail on why you believe this? |
| **COVID-19** | - How has the COVID pandemic affected your feelings on the plan to switch to a digital standardised CIMS. |
| **Perception of cohesion & involvement in change processes** | - Reflecting on your past experiences, how involved have you felt in change processes in this organisation? what has made you feel this way… e.g. of good/bad - Thinking forward to a future implementation plan, in what way would you hope to be involved in the roll out and adoption of this new method of CIM? - In the future, do you have any suggestions on how to approach bringing in a change more effectively in this organisation? |
| **Perception of usefulness** | - Thinking into the future, what difference do you think a standardised digital CIMS will make to the work of the DoPH in health protection? - What difference will a standardised digital CIMS make to your role as ­­­­­­­­­­­­­­…..? |
| **Anything else** | - Is there anything else you would like to add that should be considered for the future users CIMS during its implementation and utilisation? - Is there anything else this organisation should consider for future digitalisation projects? - Is there anything in the existing organisation that can be leveraged to increase the likely success of this implementation and or make people feel more willing to make the change? |
