# Supplementary material for: A snapshot on a journey from frustration to readiness–A qualitative pre-implementation exploration of readiness for technology adoption in Public Health Protection in Ireland
Source: PLOS Digit Health. 2024 Mar 5;3(3):e0000453. doi: 10.1371/journal.pdig.0000453 (PMC10914281; doi:10.1371/journal.pdig.0000453)
Supplement: S1 Table — (PDF) [file pdig.0000453.s003.pdf]

S1 Table. Perceptions of change from past experiences

Themes & sub-themes	Quotes
**Experiences of change in the DoPH**	
Positive	
Changes made in response to staff needs	*'There's been … local changes because we have brought up our, you know, we've identified something that needs to change and it has changed.' (E)*
Good engagement	*'we have been involved, … I think the department, … it has been very engaging with all it's staff changes on pages, so we have to move office and I think … while our own was being done up there was very good … communication done with that. There was a representative from each … grade or position, peer group, peer group. That worked very well. (H)*
Open communication	*'there is a good pathway you know. All the way up to [the Director] it's there, you know you can make your way up to her with information if you need to.' (E)*
Negative	
Not feeling part of a team	*'I definitely don't feel part of a team and I think a team approach definitely would be. It's really important to look at if they want to successfully implement [the CIMS] (D)*
Ad hoc communication & engagement	*'communication is poor. … it's not that people don't want to share information or knowledge, but it's just the process really isn't there or hadn't been there or it's a bit ad hoc. So if there's a meeting … whoever is there is … getting it [the communication/information] … It's not then being fed downstream … And again, … during the pandemic there was so much other stuff going on that you nearly did rely on … word of mouth … Because the volume of emails that were coming … you just couldn't stay on top of them. (C)*
**Experiences of change outside the DoPH**	
Positive	
Feeling engaged & part of a team	*'we're very supportive of each other. We listen to each other, which I think is, you know which … really good, nobody is afraid to thrash out ideas or to … unpick someone else's idea without it being seeing as you know a negative thing. So that like that's good.' (F)*
Early communication of plans	*'All of that was decided, you know, at the outset …, the responsibilities … all of that was … part of the agreement plan, that it was all pre agreed and sorted' (C)*<br><br>*'we were well prepared from the minute the budget say came out. We were prepared … These were going to be the changes on it. They were signed off by [leadership role]. We didn't have any choice. They were going to be coming in. But we were very well prepared with the circulars that related to that and the dates that we're going to be updated because … there was a legal obligation that they kicked in. We didn't have any choice in the matter. They made the rules, we kicked in, but we were prepared with that and at the same time … But I would think it was rolled out very well in the circulars were given.' (D)*

Negative	
Lack of consultation - change happens to end users	*'then when change happened it just happened to us without consultation. So I would say change experience hasn't been that positive, even if the change is positive … Even when the change is positive, you just find out that it's happening without being aware through the process.' (B)*<br><br>*'I think again having been part of rollouts of other systems, I think having a [role]'s input is huge because [role]s are often the ones that will use it more and they're rarely asked about it, when they're going through it. So it's just given, we are end users immediately. We were never part of the process. Well, rarely have been part of the process. So I think that's a good idea.' (E)*
Insufficient time for training	*'But usually that's because we're too busy to learn them. We actually learn them while we're using them. I suspect here that would be slightly different [here] because we're [a] smaller team.' (E)*
**Experiences of change during COVID-19**	
Good change takes time & effort	*'there was a team member of mine wanted to bring in [a practice] … it was a long process and a long constant consultation process and brought them in but [what] was really positive was the communication around it. So lots of communication before it was implemented, lots of warning before it started. And then lots of examples and kind of workshops on how to use it before it started and then lots of support after the initiative began and it … had a pilot initiative, then got feedback. The [practice] was adapted based on the feedback before it got disseminated throughout the [site]. And it's been extremely successful. And they also had baseline measurements of how it was going before and then measurement afterwards and overall the entire [site] felt it was a positive improvement and I think that whole process though probably took between two and three years, that it can be quite long.' (B)*
Rapid unorganised change during pandemic was abnormal	*'we just took it as it came. You know, … you felt that you were part of something that was happening quickly rather than with finesse. And you accept that, but you would expect then that … it would start coming together better once that real panic is over.' (E)*<br><br>*'maybe there was so little change for so many years and then there was very, I mean, you would never call it above rapid, mega rapid … maybe it … was a [inaudible] huge on social change very quickly. You know, this wouldn't be normal for most change scenarios either.' (D)*
Pre-COVID change was slow	*'I think the things that have plodded are things that were probably initiated before COVID' (F)*
