# Supplementary material for: A snapshot on a journey from frustration to readiness–A qualitative pre-implementation exploration of readiness for technology adoption in Public Health Protection in Ireland
Source: PLOS Digit Health. 2024 Mar 5;3(3):e0000453. doi: 10.1371/journal.pdig.0000453 (PMC10914281; doi:10.1371/journal.pdig.0000453)
Supplement: S2 Table — (PDF) [file pdig.0000453.s004.pdf]

S2 Table. Perceived weaknesses of CIM practice

Sub-themes	Codes	Quotes
Poor data management	• Difficult to find & extract data<br>• Ad hoc methods of storing & sharing data<br>• Data duplication<br>• reliant on frequent users to help find the data<br>• Reputational risk if files cannot be found	*'we could open up an outbreak case, a situation created and then two days later, end up opening it up, creating a whole new one for the same situation because we couldn't find the original one because … a search function wasn't clever. Or … somebody misspelled something' (E).*
Inefficient	• Time consuming<br>• Backlog of cases & delays in reporting<br>• Handover of cases challenging<br>• Human dependent input	*'it's time consuming because you have to contact a number of people. And everybody … has their own variations of how they store things ... emails, on shared drives ... just messy' (A).*
Systems outdated & not fit for purpose	• Error prone & corruptible<br>• Too many disjointed systems<br>• Not reliable<br>• Cannot cope with demand<br>• Lack of integration with other systems/databases<br>• Not user friendly	*'at the moment they come in multiple different work streams, they're not integrative, … there's paper notifications, a lab system to look at, and then [the] CIDR as well ... if you want to throw COVID into it, it's another work stream as well' (B).*
Cyber-security issues	• Cyber-attack vulnerabilities<br>• Historical back up issues	*'when the cyber-attack happened last year we were very vulnerable to that … there was … no backup to that when our systems were down for … was it almost six weeks at that time. We had to revert to paper … we didn't know if everything was going to be deleted or not … so yeah it was quite a challenge' (H).*
Doesn't facilitate teamwork	• System designed for single user	*'if one person has it open, then it's read only to everybody else … So that does cause … a lot of emails going around. "Can you close out the log? Can you close out the log?"'(F).*
Legal & privacy issues	• Potentially challenging to perform data removal requests<br>• Unclear if hard copies being kept to facilitate any investigations<br>• Challenging to get & sort information for FOI requests<br>• Challenging to audit<br>• Privacy concerns in CIM	*'you're relying on the person that's entering the information, to … sign it, so you know who's responsible for that information … the system … it's not really audited … and the ability to audit it … wouldn't be straightforward' (C).*
Lack of readily available IT support	• Reliant on national IT support service<br>• No on-site IT expertise	*'if we have a problem … with our IT system … there is no IT person on site, so that … logging our issue with the central IT service, now, they … are quite quick to respond. But … that can take some time as well, and you can lose a couple hours in the day if you're if you're willing, to act on something' (H).*
