# Supplementary material for: A snapshot on a journey from frustration to readiness–A qualitative pre-implementation exploration of readiness for technology adoption in Public Health Protection in Ireland
Source: PLOS Digit Health. 2024 Mar 5;3(3):e0000453. doi: 10.1371/journal.pdig.0000453 (PMC10914281; doi:10.1371/journal.pdig.0000453)
Supplement: S3 Table — (PDF) [file pdig.0000453.s005.pdf]

S3 Table: Perceived strengths of CIM practice

Subthemes	Codes	Quotes
Functioning digital service	• The job is being done<br>• Alternative routes to access files (when system is down)<br>• IT infrastructure & support available<br>• All COVID cases in one place<br>• Electronic system<br>• Transparency<br>• Sense of responsibility for cases	*'at least, everything is filed electronically, and … we don't have large … paper systems and filing cabinets … paper trail is now an electronic trail … so that's a good thing and it leads to probably more transparency' (H).*
Acceptable to users	• Familiarity with methods<br>• Easy learning & training<br>• Simple system<br>• Clear role definition<br>• Enables remote working	*'There's … not many steps to find where you need to be, which sometimes can be a problem learning something new. So that was beneficial because we were learning the job quickly, in real time … there was no … time to settle in' (E).*
Ownership of system		*'[the OMS is] something that was created … in house too … which is nice' (F).*

CIM - case and incident management; OMS - outbreak management system for COVID-19
