# Supplementary material for: A snapshot on a journey from frustration to readiness–A qualitative pre-implementation exploration of readiness for technology adoption in Public Health Protection in Ireland
Source: PLOS Digit Health. 2024 Mar 5;3(3):e0000453. doi: 10.1371/journal.pdig.0000453 (PMC10914281; doi:10.1371/journal.pdig.0000453)
Supplement: S4 Table — (PDF) [file pdig.0000453.s006.pdf]

S4 Table. Positive but frustrated attitude with negative perceptions of others' attitudes

Themes & sub-themes	Quotes
**Positive but frustrated attitude with negative perceptions of others' attitudes**	
Positive	
Want it to happen soon	*'I think if anything, people are probably getting frustrated that it's taking so long' (A).*
Understanding good solution & implementation takes time	*'there's no need to rush it. Obviously, you want it to be timely, but … the HSE are probably not going to spend money on something like this for a very long time. So this needs to be as good as it can be. Before it's locked down and becomes what we use. So I think … if you give time to that, it's worth it in the end' (E).*
Feeling eager, excited & looking forward to new system	*'I really think that … people are excited about it' (F).*
Confidence in leadership	*'I knew it was going to be good. And I felt like they've put so much time into it … that I knew [it] was going to be something … that has been really … thought out' (F).*
Understanding the need for a solution	*'I suppose it's an opportune time because if this happened before the pandemic … who'd know about what our needs would have been. But now we know. We've a greater depth of knowledge. We kind of have a greater understanding of what we might need in response to another scare, like a global pandemic' (G).*
Expectation the system will be good	*'because people were just hearing about the software and that it's going to be a game changer' (G).*
Expectation colleagues won't resist change	*'I think as a team … we'd be receptive to … taking on board this, as long as it makes sense' (E).*
Negative	
Feeling anxious, daunted, even fearful	*'I think anxiety around the whole system can be a problem' (E).*
Challenging due to age & digital technical ability	*'we're all different ages and different technologically abled, and I think … some people find it very easy and … some people find it a bit more challenging so support to hand would be good' (H).*
Expectation of resistance to change	*'You'll have … staff saying, "Oh well, you know CIDR still works. Do we have to?" … they might be a bit reluctant and again if it's not going to be an all or nothing approach on the diseases, then there's … other concerns then on "well … I don't want to be running two systems" … and I think that's key for us (C).*

CIDR: Computerised infectious disease reporting (system)
