# Supplementary material for: A snapshot on a journey from frustration to readiness–A qualitative pre-implementation exploration of readiness for technology adoption in Public Health Protection in Ireland
Source: PLOS Digit Health. 2024 Mar 5;3(3):e0000453. doi: 10.1371/journal.pdig.0000453 (PMC10914281; doi:10.1371/journal.pdig.0000453)
Supplement: S5 Table — (PDF) [file pdig.0000453.s007.pdf]

S5 Table. Perceived user & service benefits

Themes & sub-themes	Quotes
**Perceived user & service benefits**	
Benefits for user	
More efficient & less frustrating work	*'it will just make it more efficient. As I said, less frustrating. And I think also … if everything you need is there, you're going to be better prepared for what you're doing' (E).*
Easier management of case handovers & involvement of new staff on team	*'less time spent … repeating stuff especially at meetings where if somebody wasn't in the previous day they have to be brought up to speed because … there's no way else of getting all the information' (A).*
Benefits for public service	
Better care for patients	*'it'll allow for better, long term, for better care … for patients' (A).*
Better outbreak management through connected DoPHs	*'it's important to be able to see … not just to work in our own regions and just be looking at our own numbers and of … illnesses and notifications and stuff … you have to think nationally as well. I think if there is better linkages across the country, then … it'll be better for … the public health as a whole' (H).*
Improved data governance & accountability	*'It will be [that] you can really … stand over it … if it's designed in such a way you will be able to see stuff … I think [it/that] would be hugely beneficial (C).*
Integrated data for evidenced based PH	*'So we need the data to act and do our job. Good data, integrated data. The data that integrates with other systems … it should integrate with our HIQA system. It should integrate with our HIPE system, should integrate with our PCRs scheme or immunization. We should have a one stop shop that integrates across and that'll give us better data on our population and our populations health and that'll inform our practices' (B).*
More timely service	*'hopefully it'll allow us to identify … earlier … numbers of illnesses … clusters or outbreaks that might be happening' (H).*
Opportunity for out of hours cover	*'better opportunities to organize out of hours cover and … being able to cover for each other … expertise, being able to be got from other areas easily enough … once they've access to the system' (A).*
Cost efficiency	*'everything where there's a human cost, there's a huge financial cost and investments are obviously not just about money, but also about saving lives too' (G).*
Other benefits	
Data uses outside of CIM	*'public health research data could be...brought up for local council meetings, particularly to improve things'(D).*
Sustainability	*'that's it grows with our service too and with our … evolving needs and the evolving nature of our communities and that … it's fit for the purpose that it's intended for but that it just will grow with us and our service and our communities' (F).*

HIQA - Health Information and Quality Authority; HIPE - Hospital In-Patient Enquiry (system); PCR - Polymerase chain reaction (molecular disease testing)
