# Supplementary material for: A snapshot on a journey from frustration to readiness–A qualitative pre-implementation exploration of readiness for technology adoption in Public Health Protection in Ireland
Source: PLOS Digit Health. 2024 Mar 5;3(3):e0000453. doi: 10.1371/journal.pdig.0000453 (PMC10914281; doi:10.1371/journal.pdig.0000453)
Supplement: S6 Table — (PDF) [file pdig.0000453.s008.pdf]

S6 Table. Uncertainty about the technological solution

**Themes & subthemes**	**Quotes**
**Uncertainty about the technological solution**	
Uncertainty in CIMS specifications (technology readiness)	
Level of integration	*'Integrating into hospital systems … just that integration component, integrating it into a European system, so that … it's built in a way that has its flexibility because a lot of the systems currently in use in the HSE don't have that' (B).*
Will it facilitate remote working?	*'if I'm working remotely can I access it easily remotely from my work laptop and you know that sort of thing' (H).*
Privacy	*'To ensure that [information] was kept as anonymous as possible and only on a needs to know basis' (D).*
Security & backup	*'a backup system if things go wrong … storing patient information … that it's watertight' (H).*
Relevance of data field to Irish context	*'it hasn't been used in Ireland, so it would have to be tested … just to make sure … people have the right data fields … That they are actually gathering the right information because if they're not, they may need to go back to the hospital or to GP to make sure to ensure they're getting the [data]' (D).*
Is it an all-in-one system?	*'it really has to be clever and have everything that we need there … So that we can get rid of all the other old cobbled together stuff' (E).*
Other: Inclusion of historical data; Feedback mechanism for errors; Speed of national system	*'there's over 20 years of data on each disease … in the system. So I think we would need to know can we still access that. [those] trend lines are important to us' (C).*
Other uncertainties	
Implementation	*'I don't [know] when they're hoping to implement it, but we could be working on … a test database up to a year before things kick in' (D).*
Evaluation	*'Two factors you could consider there would be … the amount of times that maybe CIDR maxed out on the number of notifications, particularly in peaks. [And] then potentially the delayed notification … rates. I don't know if that's an easy thing to pull, but that would be something' (B].*
Consequences for local or national systems	*'I know the national data will go through HPSC, I think in this case. But data maybe also required on a local basis, so I don't know will there … be any knock on for that?' (D).*

HSE – Health Service Executive; CIDR - Computerised infectious disease reporting (system); GP – general practitioner (physician); HPSC – Health Protection Surveillance Centre
