# Supplementary material for: A snapshot on a journey from frustration to readiness–A qualitative pre-implementation exploration of readiness for technology adoption in Public Health Protection in Ireland
Source: PLOS Digit Health. 2024 Mar 5;3(3):e0000453. doi: 10.1371/journal.pdig.0000453 (PMC10914281; doi:10.1371/journal.pdig.0000453)
Supplement: S7 Table — (PDF) [file pdig.0000453.s009.pdf]

S7 Table. Barriers & enablers to readiness & willingness to adopt a CIMS

Themes & subthemes	Quote
**Barriers to readiness & willingness to adopt a CIMS**	
Barriers	
Lack of knowledge & familiarity with the system	*'because it's for everyone there, these are all going to be the end users … and to be given a product that they know nothing about or don't really know how it works, or, you know, is it going to serve their needs? You know, it's a bit crazy' (C).*
PH Reform & COVID brought a lot of change – familiarity facilitating change	*' … because this old system has been here so long that people … know how it works, know what to do and that helps facilitate roles as we … change into team working [Reform]. And so it's not a barrier to learn something new, to join a team and work in the team because people are familiar with the old system' (B).*
Added burden of learning & using a new system	*'if people are already under pressure or hassled … .you might just feel like it's another hassle' (E).*
Enablers	
Newness of workforce	*'we're still fresh enough. We're still not embedded in our current systems … We're still learning … We're still being shown how to do things … I think we're still at that stage where we're not … comfortable. So you're not rocking any boats when you come in here with something new' (E).*
Existing culture of learning	
Want uniform & connected CIM solution for better PH	*'from our perspective there is a little frustration that we're not all doing the same thing. We are such a small country. It makes no sense that we're all disjointed in this way. So I would think that that's something that's been observed by the other teams too' (E).*
Want to do something new (COVID-unrelated)	*'Eager and anxious to be doing non-COVID work for a change' (A).*
Recognition that change is necessary	*'the recognition that the system that's in place isn't ideal or optimal … We realize that … there must be better ways to capture the information that we're capturing and so yeah I think it's out of a necessity really that people are ready' (H).*
PH associated with culture of change	*'a lot of people who moved to public health have seen it as a department that's expanding and growing … [there's] opportunities here and it's ready to change' (A).*
COVID showed change can happen & rapidly	*'if we were to take anything from COVID as healthcare workers … it has made us really even more adaptable than we … were. But that … huge change can happen quickly … if everybody's on board and … [it] just has to be done' (F).*

PH- Public Health; COVID – Coronavirus disease
