# Supplementary material for: A snapshot on a journey from frustration to readiness–A qualitative pre-implementation exploration of readiness for technology adoption in Public Health Protection in Ireland
Source: PLOS Digit Health. 2024 Mar 5;3(3):e0000453. doi: 10.1371/journal.pdig.0000453 (PMC10914281; doi:10.1371/journal.pdig.0000453)
Supplement: Attachment — Submitted filename: Reviewer-Comments.docx [file pdig.0000453.s010.docx]

# **Reviewer**

**Manuscript Number:** PDIG-D-23-00266
**Title:** A snapshot on a journey from frustration to readiness – a qualitative pre-implementation exploration of readiness for technology adoption in Public Health Protection in Ireland.

**General comment:**

The study was done on an important public health issue, as Ireland is still in a plight of pervasive technology adoption in public health. The **title** is really great; it is descriptive and explanatory, and it also fits or goes with the scope of **PLOS Digital Health**. However, the manuscript has too many caveats to be considered for publication as it stands now.

**Specific comments**

I. **General Comment:**
II. **Abstract:** Your abstract is really good and clear, as we know the abstract part is the most readable part by many researchers and other parties. So your abstract must be clear and reflect your entire document content, like the **introduction,** aim or **objective, method, result** or finding**,** and **conclusion**, in a well-structured and precise way. So I advise you that it is better if you write in a well-structured way. As I try to read your abstract, all of the content is there, and I can understand which parts are introduction, aim or objective, method, result or finding, and conclusion. Someone who is not an expert may not understand which parts are **introduction**, **aim** or objective, **method**, **result** or finding, and **conclusion** since you are following an unstructured abstract.
III. **Methods and materials:** In this section, you should have clearly discussed the following sub-heading: Study design, study area and period, source population and study population, Sample size determination, study variables (like dependent or outcome variables), Operational definition, Data quality assurance, data processing and analysis, and data collection—all of this must be clearly discussed in this section.
IV. Does **Techniques to enhance trustworthiness** and **Data quality assurance** is similar
V. **Results**: good
VI. Another main point you did not consider or report on your document:
1) **conclusion**
2) **Limitation and strength of the study**
3) Declarations
   a. **Ethics approval and consent to participate**
   b. Competing interests/ Conflicting interests
   c. **Consent for publication**
   d. Funding
   e. **Availability of data and materials**
   f. Acknowledgements
   g. **List of Abbreviations**

Why you are not considering all in read on your document? Do you have any special reasons?

And check PLOS Digital Health manuscript submission format the correct please for Ethical considerations.
