# Supplementary material for: A snapshot on a journey from frustration to readiness–A qualitative pre-implementation exploration of readiness for technology adoption in Public Health Protection in Ireland
Source: PLOS Digit Health. 2024 Mar 5;3(3):e0000453. doi: 10.1371/journal.pdig.0000453 (PMC10914281; doi:10.1371/journal.pdig.0000453)
Supplement: Attachment — Submitted filename: Response to Reviewers.docx [file pdig.0000453.s011.docx]

Public Health Unit

School of Medicine

Faculty of Education & Health Sciences

University of Limerick

Co. Limerick

Ireland

6 December 2023

Dr. Yuan Lai

Academic Editor

PLOS Digital Health

PLOS

The Bradfield Centre

184 Cambridge Science Park

Milton, Cambridge CB4 0GA

United Kingdom

***Ref: Response to reviewers on Manuscript PDIG-D-2300266***

Dear Dr Yuan Li and Reviewers

Thank you for accepting our research article titled “A snapshopt on a journey from frustration to readiness – a pre-implementation qualitative exploration of readiness for technology adoption in Public Health protection in Ireland” for consideration for publication in PLOS Digital Health. We are particularly grateful to the reviewers for their time and effort in peer reviewing our manuscript. The comments and recommendations are highly appreciated and appropriate and we believe the resulting revisions have improved the manuscript greatly, particularly the readability and ease of understanding of our work.

We have responded to the reviewers’ comments below (in table format) and noted the corresponding changes in the manuscript according to revised line number. A tracked changed version of the manuscript accompanies this letter with changes/additions additionally highlighted in yellow to facilitate review. However, the line numbers refer to the revised manuscript without track changes or the ‘simple markup’ track changed document.

We thank again the reviewers and the editor for their careful consideration of our manuscript and hope that the changes and additions meet the expectations of the reviewers and editor. We found the review very helpful and the suggestions by the reviewers have greatly improved our manuscript. It is much appreciated by the group of authors.

Yours sincerely

Thea Tilley (D.O Tilley)

**Table 1: Author response to reviewer 1 comments**

|  | **Reviewer 1 comments** | **Author response** | **Line #** |
| --- | --- | --- | --- |
| ***1*** | ***General comment:***  *The study was done on an important public health issue, as Ireland is still in a plight of pervasive technology adoption in public health. The* ***title*** *is really great; it is descriptive and explanatory, and it also fits or goes with the scope of* ***PLOS Digital Health****. However, the manuscript has too many caveats to be considered for publication as it stands now.* | Thank you to the reviewer for their kind comment on the title of our work. In making the changes suggested by both reviewers we hope we have addressed the caveats noted by the reviewer. |  |
| ***2*** | ***Abstract:*** *Your abstract is really good and clear, as we know the abstract part is the most readable part by many researchers and other parties. So your abstract must be clear and reflect your entire document content, like the* ***introduction,*** *aim* *or* ***objective, method, result*** *or* *finding****,*** *and* ***conclusion****, in a well-structured and precise way. So I advise you that it is better if you write in a well-structured way. As I try to read your abstract, all of the content is there, and I can understand which parts are introduction, aim or objective, method, result or finding, and conclusion. Someone who is not an expert may not understand which parts are* ***introduction****,* ***aim*** *or objective,* ***method****,* ***result*** *or finding, and* ***conclusion*** *since you are following an unstructured abstract.* | Thank you to the reviewer for this suggestion to make our abstract more clear to the readership by making it structured. The abstract has been converted to a structured abstract, with inclusion of introduction, aim, objective, method, findings and conclusion subheadings with slight modifications the aim and summary of findings to better encompass the study. | 14,  18-19, 20,  23-24,  31 |
| ***3*** | ***Methods and materials:*** *In this section, you should have clearly discussed the following sub-heading: Study design, study area and period, source population and study population, Sample size determination, study variables (like dependent or outcome variables), Operational definition, Data quality assurance, data processing and analysis, and data collection—all of this must be clearly discussed in this section.* | We thank the reviewer for their suggestions to include the listed sections in our materials and methods. To the best of our ability we have reorganized our method details in line with these suggestions. Additionally, we revisited the EQUATOR network recommended Standards for Reporting of Qualitative Research – SRQR - <https://www.equator-network.org/reporting-guidelines/srqr/> as recommended by PLOS Digital Health for the methodology section, to ensure all the necessary details for qualitative studies are reported. We have included in the supplemental materials, the SRQR checklist outlining where information is reported in the manuscript and referred to it in the methods “Study design” section (S1 File).  The subheadings are now as follows: Study design; Researcher characteristics & reflexivity; Study context; Study area & period; Study population; Sampling strategy; Study participants; Ethical issues pertaining to human subjects; Data collection; Data processing; Data analysis; Techniques to enhance trustworthiness.  We believe the headings ‘study variables’ and ‘operational definitions’ are not appropriate to the qualitative exploratory and inductive approach to this study as we sought to identify the themes or concepts based on the data rather than pre-define them before going into the analysis.  We have now included descriptions of our process for sample size determination in the ‘Sampling strategy’. Rather than a statistical approach, we took a pragmatic approach suitable for our aims and objectives. Critically, our sampling objective was to obtain diverse data rather than representative data and this was most achievable through purposive sampling for participants with the characteristics (role and seniority) that we need to present in depth data from in our investigation. This is common in qualitative research where the objective is to obtain diverse data rather than representative data (Vasileiou, K., Barnett, J., Thorpe, S. *et al.* Characterising and justifying sample size sufficiency in interview-based studies: systematic analysis of qualitative health research over a 15-year period. *BMC Med Res Methodol* 18, 148 (2018). <https://doi.org/10.1186/s12874-018-0594-7> ). If we have misinterpreted the reviewers suggestion in this regard please accept our apologies and we would like to ask for further clarification.  The data quality assurance procedures are encompassed in the “data processing” and “Techniques to enhance trustworthiness” sections. The data processing section now describes the process to ensure accuracy of the data and security of the data. “Techniques to enhance trustworthiness” section further describes a method of triangulation, by using member checking, where participants evaluated the researchers’ interpretations of their quotes and contributions to the identified themes (Birt L, Scott S, Cavers D, Campbell C, Walter F. Member Checking: A Tool to Enhance Trustworthiness or Merely a Nod to Validation? Qualitative Health Research. 2016;26(13):1802-1811. <https://doi.org/10.1177/1049732316654870>). | 124-126  117, 127, 139, 154, 159, 165, 180, 196,215, 240, 249, 266  165-175  240-245  266-270 |
| *4* | *Does* ***Techniques to enhance trustworthiness*** *and* ***Data quality assurance*** *is* *similar* | The reviewer is quite correct that there are similarities between the terms data quality assurance and techniques to enhance trustworthiness. However, our understanding is that trustworthiness can encompass and go beyond data quality assurance, for example, reporting reflexivity and transparency throughout the study (Adler RH. Trustworthiness in Qualitative Research. *Journal of Human Lactation*. 2022;38(4):598-602. doi:[10.1177/08903344221116620](https://doi.org/10.1177/08903344221116620)).  Following our revisions to the methods section according to reviewer 1’s second comment suggestions, we feel the subheading “techniques to enhance trustworthiness” is more appropriate and in line with the SRQR than “data quality assurance”.  We have also added additional steps we had taken to ensure transparency that we had not described previously, specifically the inclusion of data tables of evidence (Cloutier, C., & Ravasi, D. (2021). Using tables to enhance trustworthiness in qualitative research. *Strategic Organization*, *19*(1), 113-133. <https://doi.org/10.1177/1476127020979329>) and reference to ‘member checking’ whose process we had described but neglected to include the terminology. | 266  272-274  268-270 |
| ***5*** | ***Results****: good* | We thank the reviewer for this assessment of our findings |  |
| *6* | *Another main point you did not consider or report on your document:*   1) ***Conclusion*** 2) ***Limitation and strength of the study*** 3) *Declarations*    a. ***Ethics approval and consent to participate***    b. *Competing interests/ Conflicting interests*    c. ***Consent for publication***    d. *Funding*    e. ***Availability of data and materials***    f. *Acknowledgements*    g. ***List of Abbreviations***   *Why you are not considering all in read on your document? Do you have any special reasons?* | Our apologies to the reviewer for neglecting to address these areas in the manuscript. We have now made additions or indicated with a heading,  - conclusion,  - limitations and strengths of the study,  - declarations of ethical approval and consent to participate  - consent for publication  - availability of data and materials.  We have also included a list of abbreviations at the end of the manuscript.  We thank the reviewer for this suggestion to include a strengths and limitation section. We have now incorporated this into the new structured discussion.  We have included a declaration of the ethical approval and consent to participate at the end of the manuscript and we have further expanded on the consent process in the methods section.  We have included details of researcher approval and participants consent to have the findings disseminated. The participant information sheet expanded that this may include sharing the findings in academic journals and conferences.  We have now included a declaration on the availability of data and materials. However, due to privacy concerns and risk of identifying participants we can only provide excerpts of transcripts on request. However, we have also included in the supplemental materials tables of illustrative quotes for themes and subthemes that are approved by participants. | 710-730  660-681  695-697  198-200  699-700  198-200  702-705 |
| *7* | *And check PLOS Digital Health manuscript submission format the correct please for Ethical considerations.* | We have not found within PLOS Digital Health’s guidelines specific details for the naming format of the section concerning the ethics of the study. The SRQR guidance refers to “Ethical issues pertaining to human subjects” and thus we have renamed it as such. We have added additional details in line with the SRQR and in relation to the reviewers comments about consent to participate and publish in the methods section. | 196-207 |

|  | **Reviewer 2 comments** | **Author response** | **Line #** |
| --- | --- | --- | --- |
| *1* | *Many thanks for the opportunity to review this manuscript which I have read with interest. I have a few suggestions for ways that the authors might want to improve to aid readability and understanding of this important work.* | We thank the reviewer for reviewing our work and providing very helpful comments to improve the manuscript. |  |
| *2* | *I suggest to move the table with the number of participant earlier into the methods section to summarise the section which describes participants, page 8.* | The description of the study participants and table has now been moved to the methods section. | 180-194 |
| *3* | *I suggest the authors set out clearly in the introduction, the aims and objectives of the study and then perhaps reflect at the end of the manuscript within the discussion any reflections against original objectives.* | We have now included an explicit statement of study aim and objectives in the introduction and revisited and commented on these in the Discussion. | 98-115  569-574 |
| *4* | *The themes are nicely presented in the thematic map visual on Page 14 and throughout the results section in the for of the sub theme map visuals. However there is inconsistency between the main thematic map on page 14, the sub theme visuals and the section headings and text that follows throughout the results section. This makes the results section difficult to follow, with the visuals not flowing clearly into the sections, as well as theme headings which do not reflect the text that follows. I therefore suggest the results section would benefit from restructuring to ensure it is easy to read and flows logically from the first visual presentation of the themes and subthemes.  Ensure headings are consistent throughout and reflect themes and the sub themes as they have been presented. Start each section with the visual (perhaps with an introduction) and then ensure the subheadings and description of findings flow from the thematic visual / flow map. Furthermore, make sure all headings are describing the themes you have presented - e.g. Digital literacy is described well, but I can’t find this presented on any of the flow maps as a theme.* | We apologise for our oversight in editing our manuscript. We have reviewed our thematic map and subtheme map flow diagrams for consistency and corrected as necessary so the theme headings align between the text and the figures. As kindly suggested by the reviewer, each main category section now contains a brief introduction and a summary figure encompassing the category and resulting themes and subthemes before discussing the themes in detail in the main text.  We also included some further details in the narrative for perceived weaknesses of CIM practice so they better reflect the theme and figure – specifically that the existing practice doesn’t facilitate team work and there are issues with readily available IT support and a supporting quote (and participant approved quote). Similarly for perceived strengths of CIM practice we added detail on the nature of the system (simple and transparent, easy training and facilitation of remote working.  The section on barriers and enablers of readiness and willingness has been reorganised, moving the barriers and enablers discussed under other themes under this theme alone. We have removed the section on enabler of readiness and willingness – sense of newness, as a consequence. | 286-299  314, 337-346, 347-349, 353, 360, 377, 385, 404-416, 431, 438-440  458, 500-510, 524, 541-562  360-367  373-376  379-380  541-562 |
| *5* | *Your page 14 thematic map has arrows reflecting relationships / dependencies between themes, For example, I could imagine that past experiences of change might influence readiness and willingness to implement new technology? I would argue that there are more linkages then presented. I suggest the approach taken to identify these dependencies / linkages were identified or is it the authors opinions?*  Response:  . | Thanks to the reviewer for highlighting this. Our analysis approach did not set out to describe new conceptual linkages so to avoid suggesting so we have decided to remove these from the thematic map. We do however describe specific linkages in our results narrative that arose directly from participants explanations of why they had specific feelings or attitudes, including readiness and willingness. This reflected the questions in the interview guide. These provide the understanding for why future users feel ready or willing or not ready and willing. | This is uploaded separately in the new submission (fig 2).  542-543  547-548  557-559 |
| *6* | *Finally, I suggest you break up the discussion section with sub-headings to aid readability.* | We have structured our discussion according to summary of findings – the rich picture, theories and models of technology adoption, barriers and facilitators to adoption, opportunities for organisational change, strengths and limitations of the study, and conclusion. We thank the reviewer for this suggestion. | 575, 599, 630, 647, 660-670, 682 |

We thank again the reviewers and the editor for their careful consideration of our manuscript and hope that the changes and additions meet the expectations of the reviewers and editor. We found the review very helpful and the suggestions by the reviewers have greatly improved our manuscript. It is much appreciated by the group of authors.

Yours sincerely

Thea Tilley (D.O Tilley)
